# Extracting Subtask-specific Metrics Toward Objective Assessment of Needle Insertion Skill for Hemodialysis Cannulation

**DOI:** 10.1142/s2424905x19420066

**Published:** 2020-04-14

**Authors:** Ziyang Zhang, Zhanhe Liu, Ravikiran Singapogu

**Affiliations:** Department of Bioengineering, Clemson University, 301 Rhodes Research Center, Clemson, SC 29634, USA

**Keywords:** Vascular access, hemodialysis cannulation, medical training simulator, skill assessment

## Abstract

About 80% of all in-hospital patients require vascular access cannulation for treatments. However, there is a high rate of failure for vascular access cannulation, with several studies estimating up to a 50% failure rate for these procedures. Hemodialysis cannulation (HDC) is arguably one of the most difficult of these procedures with a steep learning curve and an extremely high failure rate. In light of this, there is a critical need that clinicians performing HDC have requisite skills. In this work, we present a method that combines the strengths of simulator-based objective skill quantification and task segmentation for needle insertion skill assessment at the subtask level. The results from our experimental study with seven novice nursing students on the cannulation simulator demonstrate that the simulator was able to segment needle insertion into subtask phases. In addition, most metrics were significantly different between the two phases, indicating that there may be value in evaluating participants’ behavior at the subtask level. Further, the outcome metric (risk of infiltrating the simulated blood vessel) was successfully predicted by the process metrics in both phases. The implications of these results for skill assessment and training are discussed, which could potentially lead to improved patient outcomes if more extensive validation is pursued.

## 1. Introduction

### 1.1. Background

Cannulation for vascular access is one of the most technically challenging and problem-ridden skills in medical care. It is estimated that approximately 80% of all in-hospital patients need to be cannulated for treatments [1]. Unfortunately, there is a high rate of failure for vascular access cannulation, with several studies estimating up to a 50% failure rate for these procedures, “in even the best of hands” [1–3]. Along these lines, the average number of attempts until successful cannulation was reported to be close to 2.5 [3]. As a result, there is a tremendous “cost” associated with cannulation errors including medical complications, patient morbidity, and financial wastage. In today’s environment of increased emphasis on patient safety by reducing medical errors, educators realize the criticality of reducing cannulation-related errors [4].

Among vascular access cannulation, cannulation for hemodialysis is arguably one of the most difficult skills [5–7]. There are unique challenges associated with hemodialysis cannulation (HDC). First, the vessels cannulated tend to have irregular shapes with large diameters since dialysis requires greater blood flow. Consequently, arteriovenous (AV) fistulas or grafts (AVGs) are recommended to be cannulated if they are larger than 6 mm in diameter. Second, the needles used in HDC are larger, ranging from 17 G to 14 G. The larger size of the needle presents the added risk of greater tissue trauma and vessel damage. Finally, since hemodialysis is performed multiple times during a week, care must also be taken not only to choose the optimal needle insertion site, but also to “rotate” the insertion sites so as to not overuse a specific location.

The steps followed for HDC are similar to other cannulation procedures: locating the blood vessel accurately using manual palpation or imaging, inserting the needle using optimal motion and force until blood enters the cannula (called “flashback”), advancing the needle forward to allow sustained blood withdrawal, and finally securing the needle [5]. Incorrectly following these steps is considered procedural error. Another serious type of error is called infiltration, where the needle punctures through the bottom or the sides of the blood vessel wall after “flashback”. Infiltration can lead to a variety of adverse medical complications that include thrombosis, scabbing, stenosis, intense pain, and even loss of a functioning vascular access [3, 8]. Since the vascular access of dialysis patients is their lifeline, errors during cannulation could lead to serious morbidity or death. It is imperative, therefore, that the clinical community work toward improving cannulation success and thus patient outcomes.

In light of this, there is a critical need that clinicians performing HDC have requisite skills. Traditionally, nurses and patient care technicians had minimal training before learning to cannulate “on the job”. For training, synthetic “fake arms” that simulate an arm with fluid through encased piping are used for teaching HDC. These rudimentary simulators do not provide objective feedback nor do they simulate the variety of anatomical/physical features commonly encountered during the process of care. During training, some educators employ checklists for rating procedural flow and technical skills, but these have limited value for skill assessment [9]. Consequently, there is a great need to create tools for structured training with meaningful objective feedback.

### 1.2. Previous work

In contrast with subjective assessment, objective sensor-based metrics have demonstrated great value in skills assessment. Some advantages of objective metrics include automatic assessment without the need for an expert supervisor [10], identifying finer aspects of skill [11], tracking skill progression [12], and learning in a low-stakes simulator-based environment. The use of sensor-based tools for medical skills assessment has gained significant attention in the last two decades. For instance, simulators with state-of-the-art sensors have been developed for laparoscopic cholecystectomy and robotic surgery [13]. For procedures involving needle insertion skills, however, relatively few studies exist. Some groups have developed virtual reality-based interfaces (including haptic devices) for training lumbar punctures as well as augmented reality systems for training spinal facet joint injections and ophthalmic surgery [9, 14–16]. For lumbar puncture assessment, electromagnetic (EM) trackers have been used in several studies to examine the motion trajectories of needles during insertion [15]. Yeo *et al*. recorded hand motion from EM trackers and noted that metrics obtained from sensor data were able to quantify skill improvement as training progressed [15]. Similarly, Clinkard *et al*. reported that quantitative needle metrics from EM data demonstrated a trend of decreased needle movement distance with more practice and experience [9]. These metrics also identified subjects who struggled with needle insertion, whereas commonly used checklists for rating skills were not able to identify this deficiency. Similarly, Chen *et al*. used an EM sensor for analyzing surgery residents’ skill during central venous catheterization (CVC) procedures [17]. Results from their study demonstrated a significant increase in the average pass rate for the procedure while significant decreases were noted in the average angle of insertion, path length, and jerk after training. Objective metrics, therefore, could be useful in assessing various aspects of skill, whether in a particular modality (e.g. motion, force) or at a certain part of the procedural workflow or at a finer granularity of skill (task versus subtask). Further, metrics can also be classified as process or outcome metrics; outcome metrics measure whether the result of a procedure is a success or failure and are closely tied to clinical outcomes, while process metrics measure skills during the course of the procedure. A more complete characterization of vascular access cannulation will include these various kinds of metrics that are domain-specific.

Automatic workflow segmentation is another aspect that has received recent attention since medical procedures are often complicated, involving multiple sequential and sometimes concurrent steps with a variety of context-specific tools. Successful outcomes in medical procedures greatly rely on whether clinicians follow the workflow accurately. Research in this field seeks to provide the context for skill assessment by identifying specific steps during medical procedures. Methods used for workflow segmentation include dynamic time warping [18], simple thresholding [19], *K*-means clustering [19, 20], Markov modeling [21], and computer vision [22]. The combination of workflow segmentation and metrics for technical skill provide procedural context-specific skill assessment.

### 1.3. Our contributions

In this work, we combine the strengths of sensor-based objective skill quantification and task segmentation for analyzing needle insertion skill at the subtask level. Whereas previous researchers have developed methods for assessing procedural flow and objective metrics at the task level, we posit that there is value in assessing metrics at the subtask level for greater precision and more efficient skill assessment [23]. That is, needle insertion, which is a part of cannulation, is further broken down into subtasks: obtaining flashback, rotating the needle, and leveling out and securing the needle. Quantifying skill using specific process and outcome metrics at this level of detail allows for more meaningful skill assessment.

Toward this, we sought to address three research questions in this work with three corresponding hypotheses:

1. *If our simulator is able to segment a cannulation procedure recorded by sensor data into subtasks (phases)*: Demonstrating that the simulator is capable of accurate segmentation of needle insertion at the subtask level is the first step towards extracting relevant metrics. H1: Our simulator hardware and software methods can successfully segment the HDC task at the subtask level.
2. *If sensor-based metrics are different in different phases*: Here, we are interested in examining if needle insertion behavior is different between two phases. If it is different, skill could be assessed and trained at the subtask level using relevant phase-specific metrics on the simulator. H2: Metrics assessed in different phases are different.
3. *If simulator-based outcome metrics are predicted by process metrics*: Since fistula infiltration is the outcome of interest in this study, if a relationship between process and outcome metrics on the simulator is evidenced, a basis for effective training could be established. H3: Simulator-based infiltration outcome metrics are predicted by process metrics in different phases.

## 2. Methods

### 2.1. Simulator platform

**Fig. 1.**
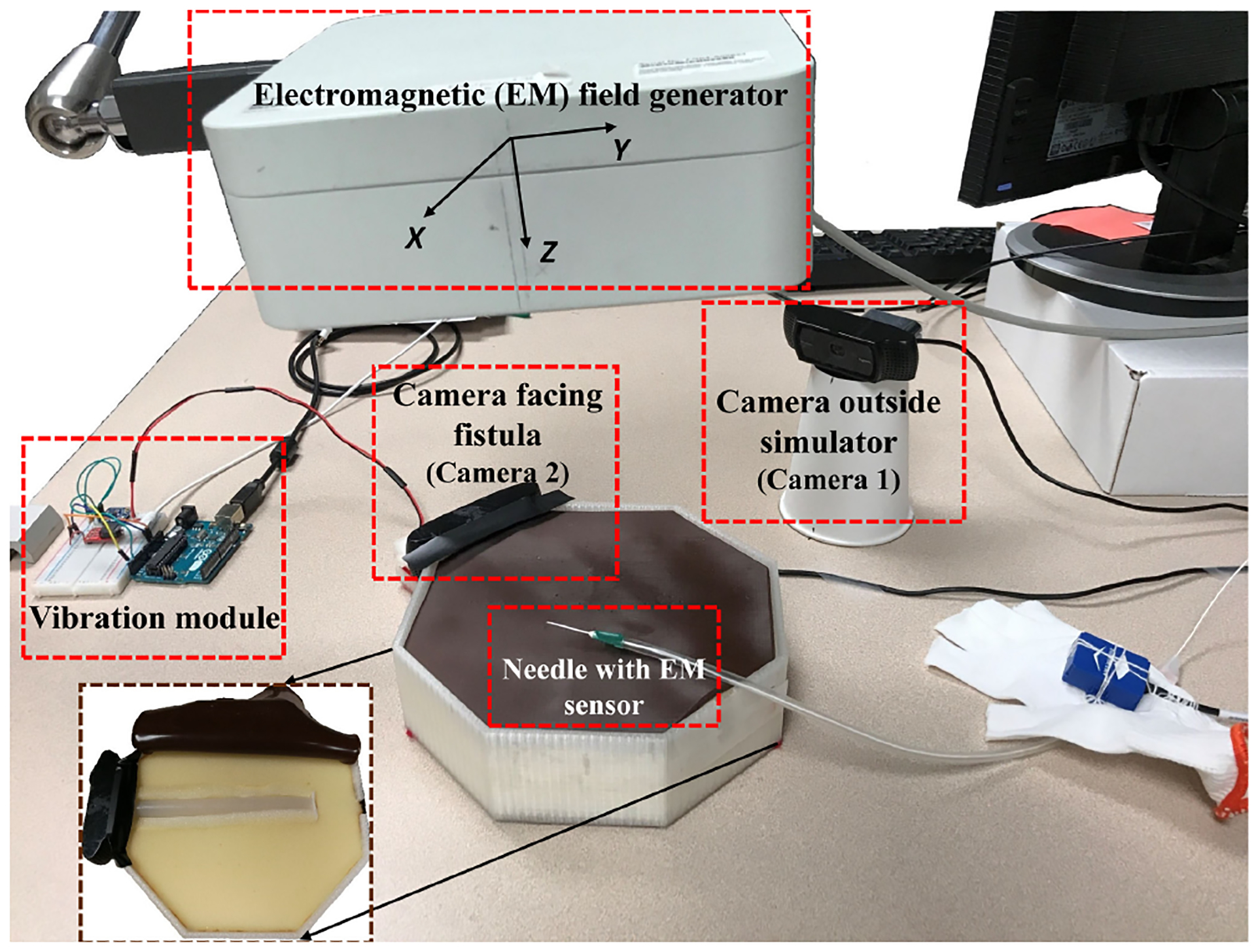
The cannulation simulator with its various components. Inset: Inside view of the simulator.

A custom cannulation simulator (see Fig. 1) was constructed consisting of a layer of artificial skin (thickness = 3 mm; Ecoflex-30, Smooth-On Inc.), beneath which an artificial straight AV fistula (inner radius = 7 mm; length = 150 mm; Ecoflex-30) was placed. A vibration motor (Model 308–107, Precision Microdrives, Ltd.) under the fistula rendered haptic feedback by converting a sample fistula bruit audio into the corresponding vibration pattern through a haptic motor controller (DRV2605L, Adafruit) and Arduino Uno. All components were fitted into an octagonal 3D-printed plastic box. An EM tracking system (Aurora, Northern Digital Inc.) was used to track the motion of the needle tip by placing a 6 DOF sensor (diameter = 1.3 mm) inside a 16 G beveled dialysis needle (Medisystems NxStage Inc.). Two cameras (Logitech C920 HD USB 2.0), one positioned outside the simulator and another positioned inside the fistula, recorded the video of the needle and hand motion during the cannulation and the video during needle insertion, respectively. The sampling frequency of the EM tracker and cameras were 40 Hz and 15 fps respectively. Three expert dialysis cannulators comprehensively examined the simulator and approved its realism and pedagogical value.

The EM tracker recorded the position and orientation of the sensor located inside the needle. The needle tip position (*P*_tip_), therefore, can be calculated using the position of the sensor (*P*_sensor_), the offset displacement of the needle tip relative to the EM sensor attached inside the needle (*d*), and a rotation matrix from the electromagnetic field coordinate frame to the sensor coordinate frame (*R*) via Eq. (1).

(1) $$P_{\text{tip}}(x,y,z)=P_{\text{sensor}}(x,y,z)+R\cdot d(x,y,)$$

### 2.2. Data synchronization

**Fig. 2.**
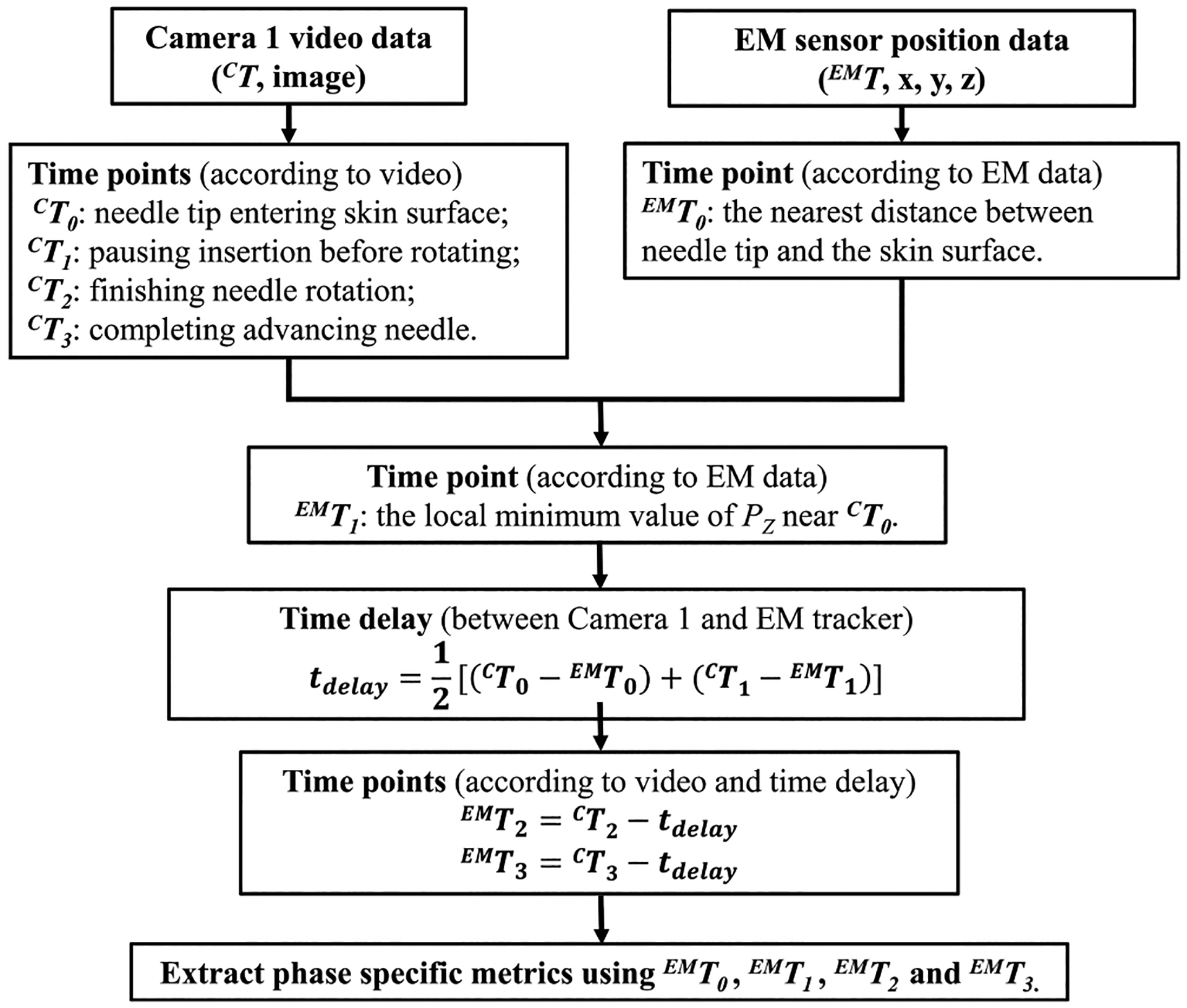
The process of cannulation workflow segmentation. *Note*: *P*_*Z*_ is the position value of needle tip along *Z*-axis [vertical direction].

Although the EM tracker recorded the motion of the needle tip during cannulation, this data alone is insufficient to segment workflow because of the variability in subjects’ individual cannulation trajectories. Hand and needle motion videos recorded by the cameras were manually inspected to determine the time points at which the four cannulation steps outlined below were initiated (see Fig. 2). To compare hand and needle motion simultaneously, custom code was written in Visual Studio C++ 2013 (including OpenCV 3.0) to synchronize EM tracker data and camera video using their respective timestamps. A Graphical User Interface (GUI) was programmed in MATLAB [24] to display the synchronized video and needle tip motion data for extracting key time points for subtask segmentation.

### 2.3. Workflow segmentation and metrics extraction

According to expert dialysis nurses [5], HDC includes the following four key steps:

*Step* 1. Identify the point of needle insertion using visual inspection and palpation of the AV fistula;
*Step* 2. Insert the needle into the fistula at a 20–35° angle with the bevel facing upward;
*Step* 3. Rotate the needle 180° to prevent back-wall infiltration after blood “flashback”;
*Step* 4. “Level out” and advance the needle for securing it.

Of the four steps mentioned above, the first pertains to determining where to insert the needle, whereas the last three pertain to the needle insertion technique itself. Consequently, for analyzing needle insertion skill, we divided phases as follows: “Insertion phase” (Phase 1), “Rotation phase” (Phase 2), and “Leveling and forwarding phase” (Phase 3), respectively. These phases are identified by four events: needle tip entering skin surface, pausing insertion before rotating, finishing needle rotation, and completing advancing the needle. The four events are simultaneously recorded by both the camera outside the simulator (Camera 1) and the EM tracker. There was a time delay between the synchronized camera video and the EM tracking data when detecting the same event, because of different sampling frequencies. It is assumed that the time delay between the camera and EM tracker is constant. The four event time points with respect to camera video (^*C*^*T*) were noted by manual inspection done by the authors during data processing. The time delay (*t*_delay_) was calculated by averaging the time differences between Camera 1 and the EM sensor at the first two events. Subsequently, the corresponding time points in the EM data stream, denoted ^EM^*T*, at the last two events were obtained by subtracting the time delay (*t*_delay_) from the corresponding time points with respect to the camera video (^*C*^*T*) to extract the phase specific motion metrics of the needle tip. The details of this methodology are illustrated in Fig. 2.

**Fig. 3.**
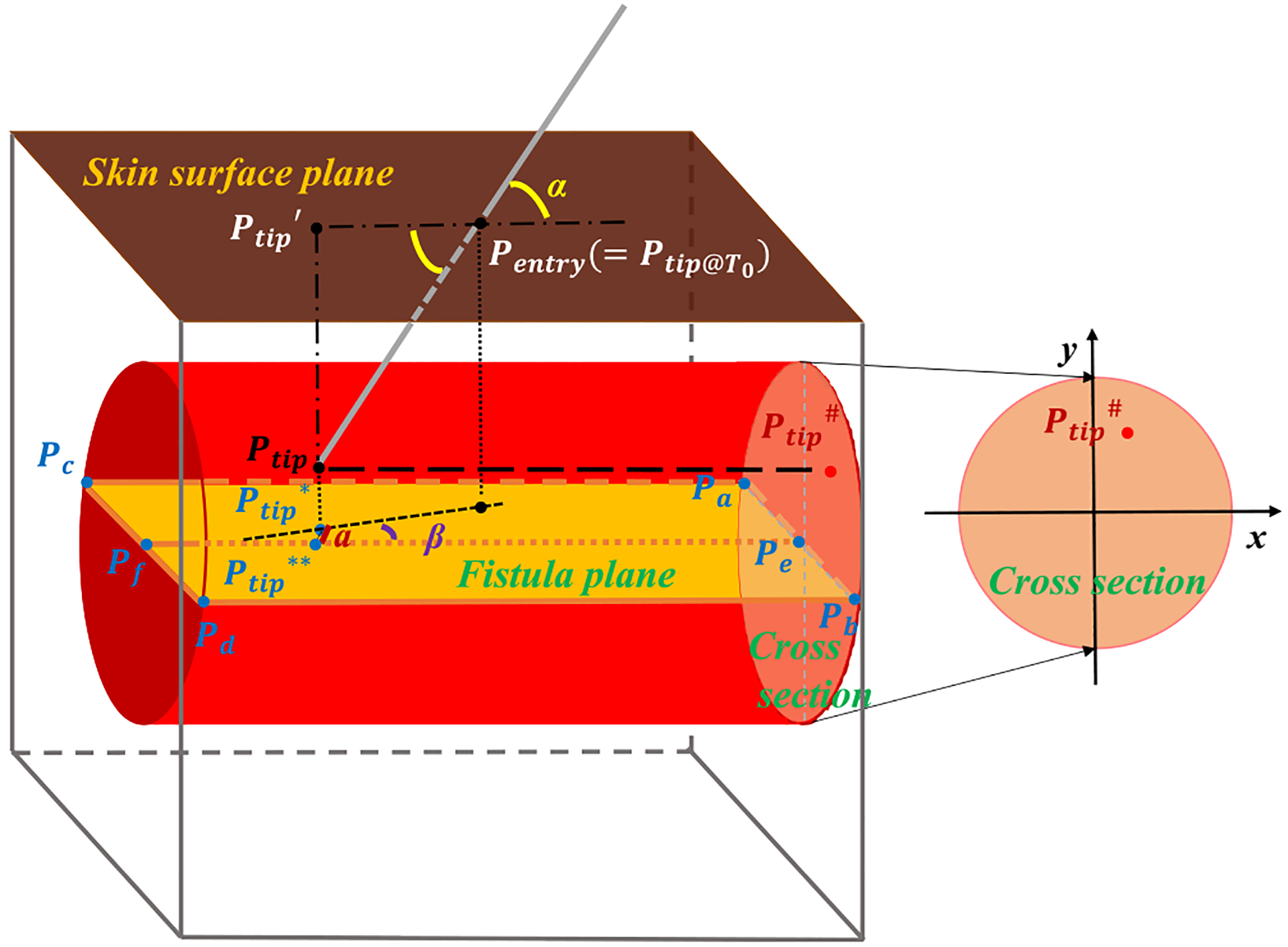
An illustration showing how planes and metrics are calculated using sensor measurements at various points of simulator geometry.

In order to perform cannulation workflow segmentation and metrics extraction, three spatial planes were defined as the skin surface plane, the fistula plane and the cross sectional plane of the fistula (see Fig. 3). The skin surface plane was determined by fitting the equation of a plane (see Eq. (2)) with several point positions measured on its surface using the EM tracker.

(2) ax+by+cz+d=0

The fistula plane was determined by fitting any three of the four measured vertices (*P*_*a*_, *P*_*b*_, *P*_*c*_, and *P*_*d*_) of its plane to Eq. (2). The axial line (*P*_*e*_*P*_*f*_) of the fistula was determined by calculating the middle points on its short sides. The cross sectional plane was calculated as perpendicular to the fistula plane with *P*_*e*_ as its center.

The needle motion metrics extracted from sensor data can be used to quantify cannulation insertion skills. In this paper, we propose two kinds of simulator-based metrics: process metrics and outcome metrics (see Table 1).

The very first step for a successful cannulation is to find the orientation of the fistula correctly through palpation. Two metrics, the start point accuracy and the lateral angle of the needle, indicate whether the location and the orientation of the fistula are found correctly during the procedure. During needle insertion, other process metrics are computed that assess the subject’s technique during cannulation, including completion time, path length traversed by the needle tip, average velocity, average insertion angle, and average rate of change of insertion angle. Simulator-based outcome metrics measure the outcome of the HDC task on the simulator, that is, whether infiltration occurs during or after the task. Two outcome metrics, infiltration risk and final needle tip position, are computed.

1. Time (*t*_*i*_). Time spent in each phase during one cannulation insertion trial. *i* denotes the specific phase (Phase 1: *i* = 1 or Phase 3: *i* = 3).
2. Path length (PL_*i*_). The total distance traversed by the needle tip during each phase. It was calculated as the summation of movement in the *X*, *Y*, and *Z* directions based on the needle tip trajectory using the Euclidian distance formula.
  (3) $${\text{PL}}_{i}=\overset{T_{i}}{\underset{t=T_{i-1}}{\sum }}\|P_{t+\Delta t}(x,y,z)-P_{t}(x,y,z)\|,$$
  where Δ*t* is the sampling period and *T*_*i*_ is the time point mentioned in Fig. 2.
3. Average velocity (*v*_*i*_). Average speed of the needle tip in each phase during one trial.
  (4) $$v_{i}=\frac{{\text{PL}}_{i}}{t_{i}}$$
4. Average insertion angle (*α*_*i*_). Average angle of the needle relative to the skin surface plane during Phases 1 and 3. It was calculated by taking the average value of the angle between the line defined by each needle tip point of the 3D trajectory (*P*_tip_) and the needle entry point (*P*_entry_) and skin surface during the specific phase.
  (5) $$\alpha_{i}=\frac{1}{N}\cdot \sum_{t=T_{i-1}}^{T_{i}}\text{arctan}\left(\frac{\left\|P_{\text{tip}}(x,y,z)-P_{{\text{tip}}^{\prime }}(x,y,z)\right\|}{\left\|P_{{\text{tip}}^{\prime }}(x,y,z)-P_{\text{entry}}(x,y,z)\right\|}\right),$$
  where *N* is the total number of sampling points of the EM tracker during one phase.
5. Average rate of change of insertion angle $\left({\dot{\alpha }}_{i}\right)$. The average change rate of needle insertion angle at Phases 1 and 3.
  (6) $${\dot{\alpha }}_{i}=\frac{1}{N}\cdot \overset{T_{i}}{\underset{t=T_{i-1}}{\sum }}\frac{\alpha_{i}(t+\Delta t)-\alpha_{i}(t)}{\Delta t}$$
6. Start point accuracy (*a*_*i*_). Distance between the projected point of the needle tip at time *T*_0_ (or *T*_2_) on the fistula plane (*P*_tip_*) and that to the axial line of the fistula (*P*_tip_**).
  (7) $$a_{i}=\|P_{\text{tip }@T_{i-1}}{}^{*}-P_{\text{tip }@T_{i-1}}{}^{**}\|,$$
  where *i* = 1 or 3.
7. Lateral angle (*β*_*i*_). Angle between the axial line of the fistula and the projected vector from the needle tip location at time *T*_*i*−1_ to the needle tip location at time *T*_*i*_ on the fistula plane $\left(\vec{P_{\text{tip}@T_{i-1}}{}^{*}P_{\text{tip}@T_{i}}{}^{*}}\right)$, where *i* = 1 or 3.
  (8) $$\beta_{i}=\text{arc}\;\text{sin}\left(\frac{\left|a_{i}-\left(\left\|P_{\text{tip}@T_{i}}{}^{*}-P_{\text{tip}@T_{i}}{}^{**}\right\|\right)\right|}{\left\|P_{\text{tip}@T_{i}}{}^{*}-P_{\text{tip}@T_{i-1}}{}^{*}\right\|}\right)$$
8. Infiltration risk (*x*_*i*_, *y*_*i*_). Projected 2D point of the needle tip position on the cross section of the fistula. For Phase 1, the position point where the value along the *Z*-axis (vertical direction) is minimum was found, and projected to the cross sectional plane to get a 2D point (*x*_1_, *y*_1_).
  For Phase 3, all the points on the 3D needle tip trajectory at Phase 3 were projected to the cross sectional plane of the fistula, and the distances between the center of the cross section and these projected points were calculated to find the 2D point (*x*_3_, *y*_3_) with maximum distance.
9. Final position of the needle tip (*x*_*f*_, *y*_*f*_). Projected 2D point of the needle tip location at time *T*_3_ on the cross section of the fistula.

### 2.4. Experimental design and protocol

After ethics approval was obtained from the Institutional Review Board (IRB), seven nursing students from Clemson University participated in the study. After receiving relevant informed consent, a questionnaire was given to each participant. The questionnaire was used to obtain information about the participants’ experience of cannulation. None of them had experience with HDC other than some intravenous cannulation experience.

Before the experiment, the subjects were asked to cannulate twice according to the standardized scripted instructions prepared to orient subjects to the simulator. During the experiment, each subject performed the cannulation task six times consecutively. All participants successfully completed all four steps of needle insertion.

### 2.5. Verification of synchronized system for workflow segmenation

**Fig. 4.**
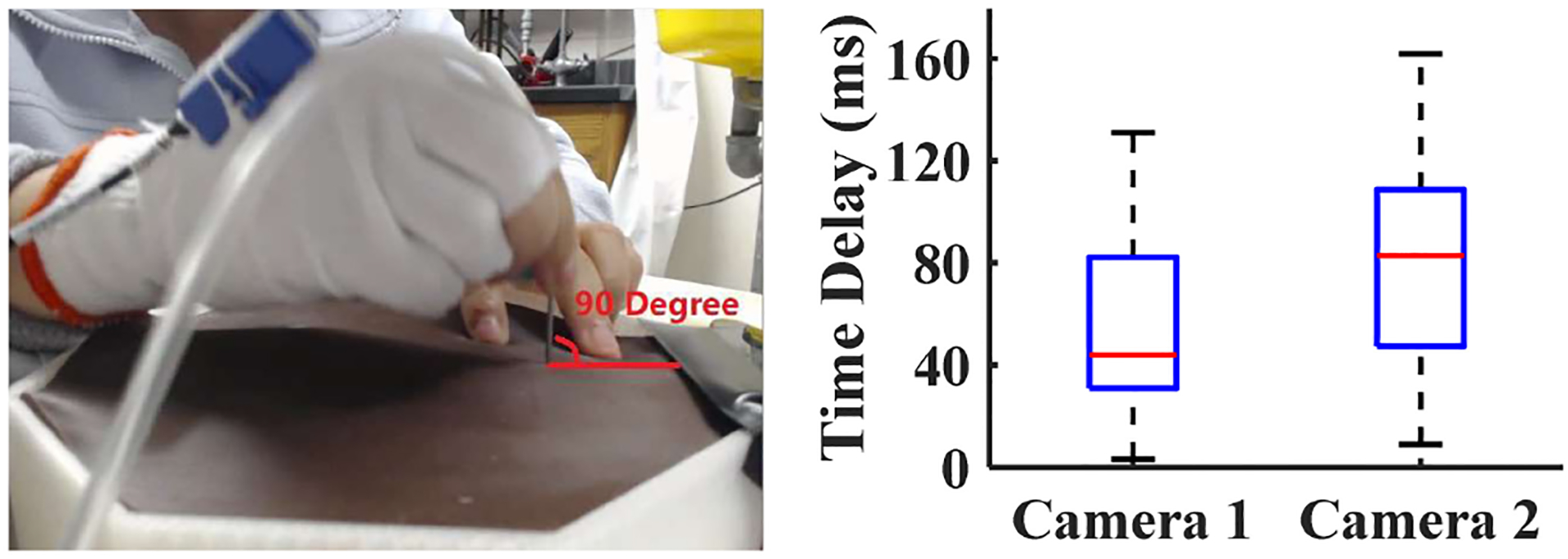
The verification test of the synchronized system with regard to the sampling rate, performed by calculating the time delays among sensors before the experiment.

**Fig. 5.**
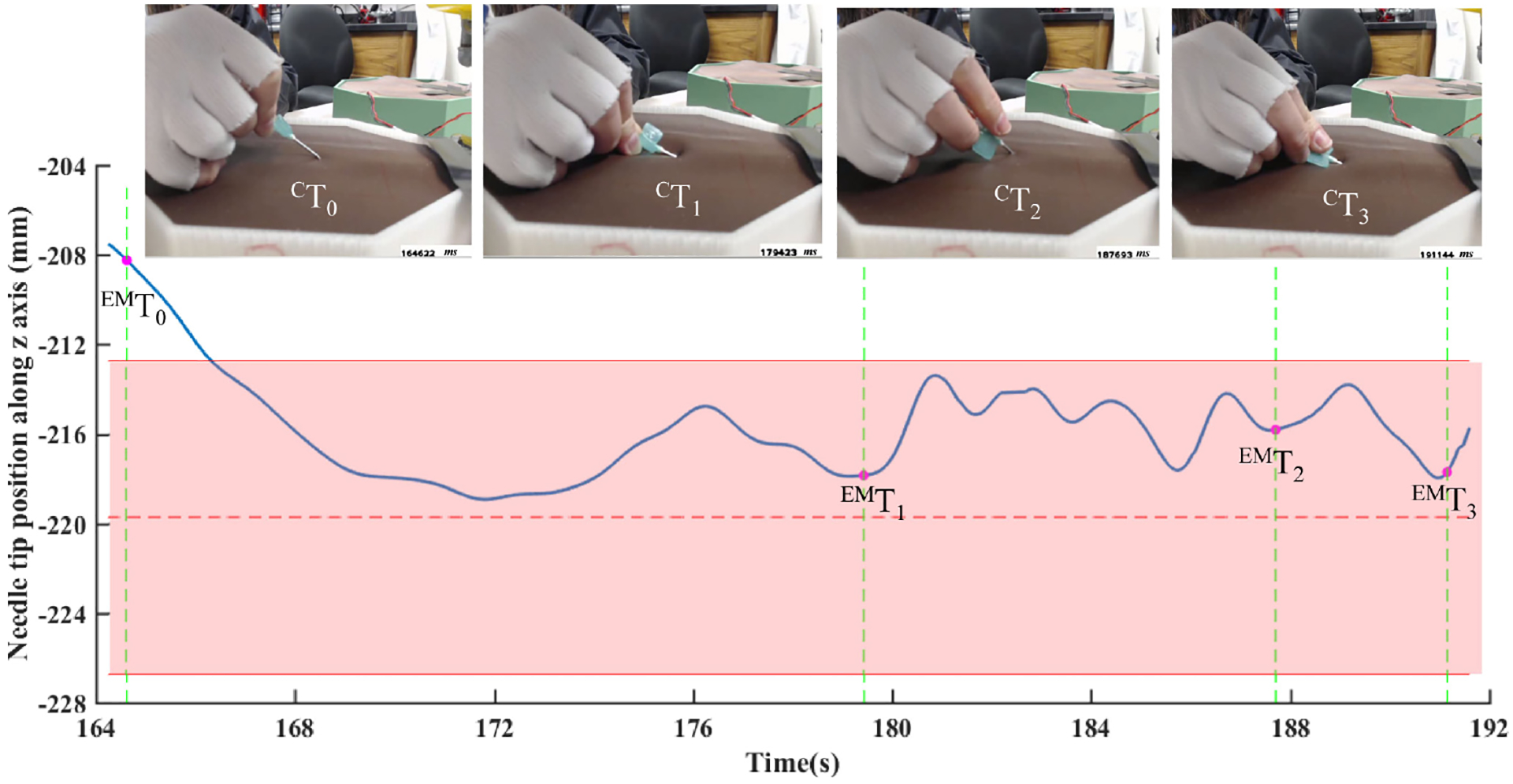
The workflow segmentation of one cannulation cycle, obtained by inspecting the cycle in MATLAB GUI and using time delay.

To verify whether the sampling rates of the EM tracker and the cameras were sufficient, a verification test, quickly pulling out the needle from the fistula after inserting it vertically was conducted before the experiment. The time delays between the EM tracker and the cameras when detecting the same event during this test is shown in Fig. 4. The maximum time delay between the EM tracker and Camera 1, which is outside the simulator, is 130 ms. In all the cannulation trials of all seven subjects, the average value of time delay (*t*_delay_), which was calculated in the way presented in Fig. 2, was 194.9 ms. Although data from each device were sampled at different frequencies, all the events before and during the experiment were detected by inspecting motion and video data in our MATLAB GUI (see Fig. 5). Therefore, by including the time delay factor we were able to observe the movement of the needle tip accurately through different types of recording devices.

**Fig. 6.**
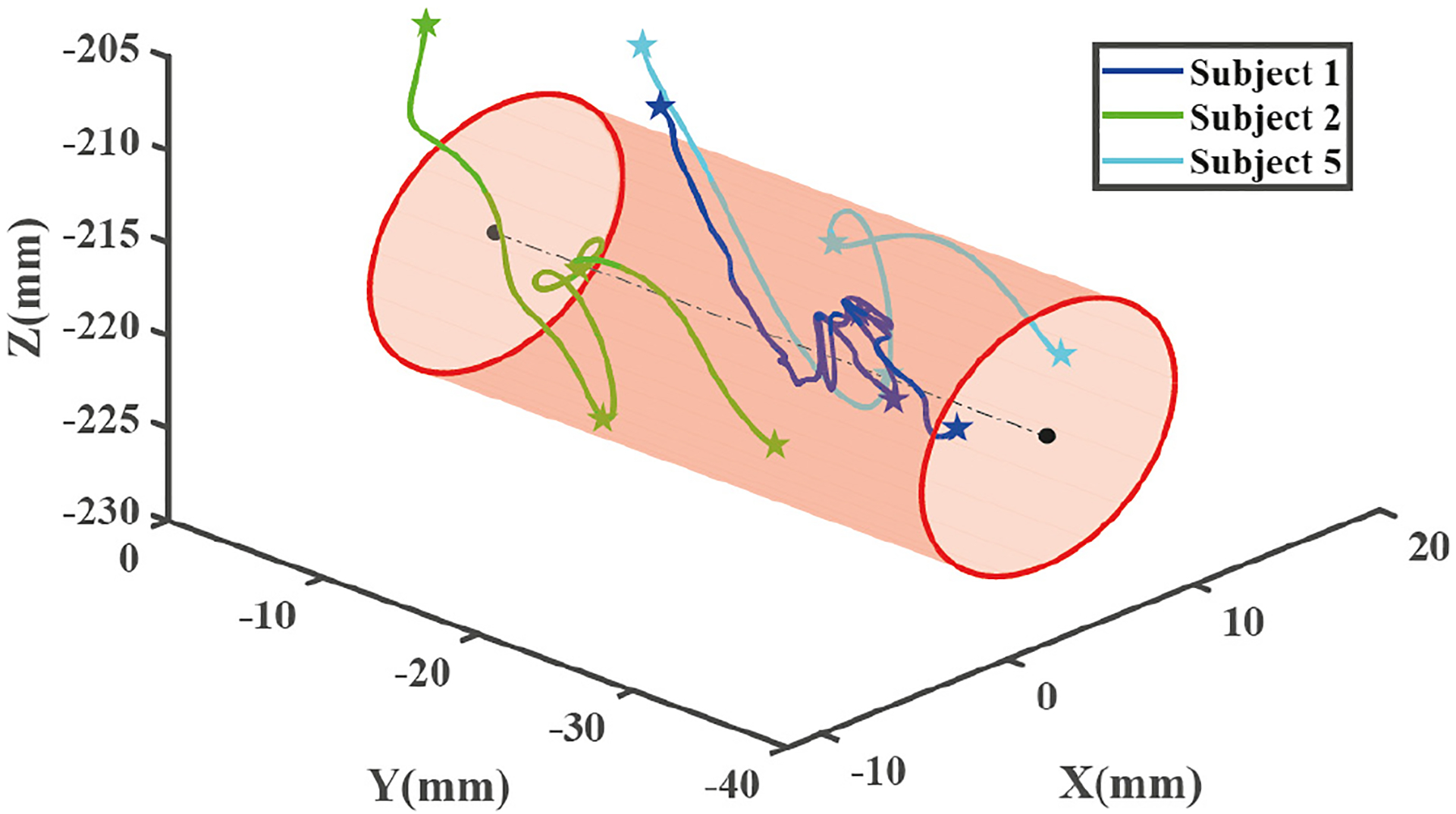
The 3D trajectory of the needle tip of three cannulation cycles from three subjects.

For each of tge cannulation procedures done by the seven subjects, the cannulation cycle was trimmed to include the needle movement from entering the artificial skin surface to the end of needle advancement. Each cannulation cycle was divided into three phases by four time points. Figure 5 shows the progression of the needle tip position in the *Z*-axis during one cannulation cycle. For example, in the first phase there are many local minima which reflect the motion of retracting and reinserting the needle, according to the video recorded outside the simulator. After checking all the trajectories of the needle tip and the camera videos during cannulation, needle motions in all three phases, including pause, retraction, and changing the insertion angle, can be reflected from the EM tracking data. Figure 6 shows the 3D trajectories of the needle tip from three trials from three different subjects with respect to the fistula volume. The stars marked in those trajectories are the four time points that were used for workflow segmentation.

### 2.6. Statistics

To answer our research questions, statistical analyses were performed using Minitab (v.18.1). To determine which statistical test was to be used, the normality of the metrics of Phases 1 and 3 was tested first. When the normality was not rejected, paired *t*-tests were used to test significant differences. Otherwise, the nonparametric Mann-Whitney *U*-tests were used. The level of significance was set to be 0.05.

For testing Hypothesis 3, a multiple linear regression model was used to study the relationship between process metrics and outcome metrics. The multiple linear regression model was constructed per Eq. (9) where each coefficient is a 2 × 1 matrix. The first element of the coefficient matrix represents the effect of a particular independent variable on the *x* value of the dependent variable, whereas the second element does so on the *y* value.

## 3. Results

### 3.1. Phase-based metrics differ in different phases

#### 3.1.1. Process metrics

**Fig. 7.**
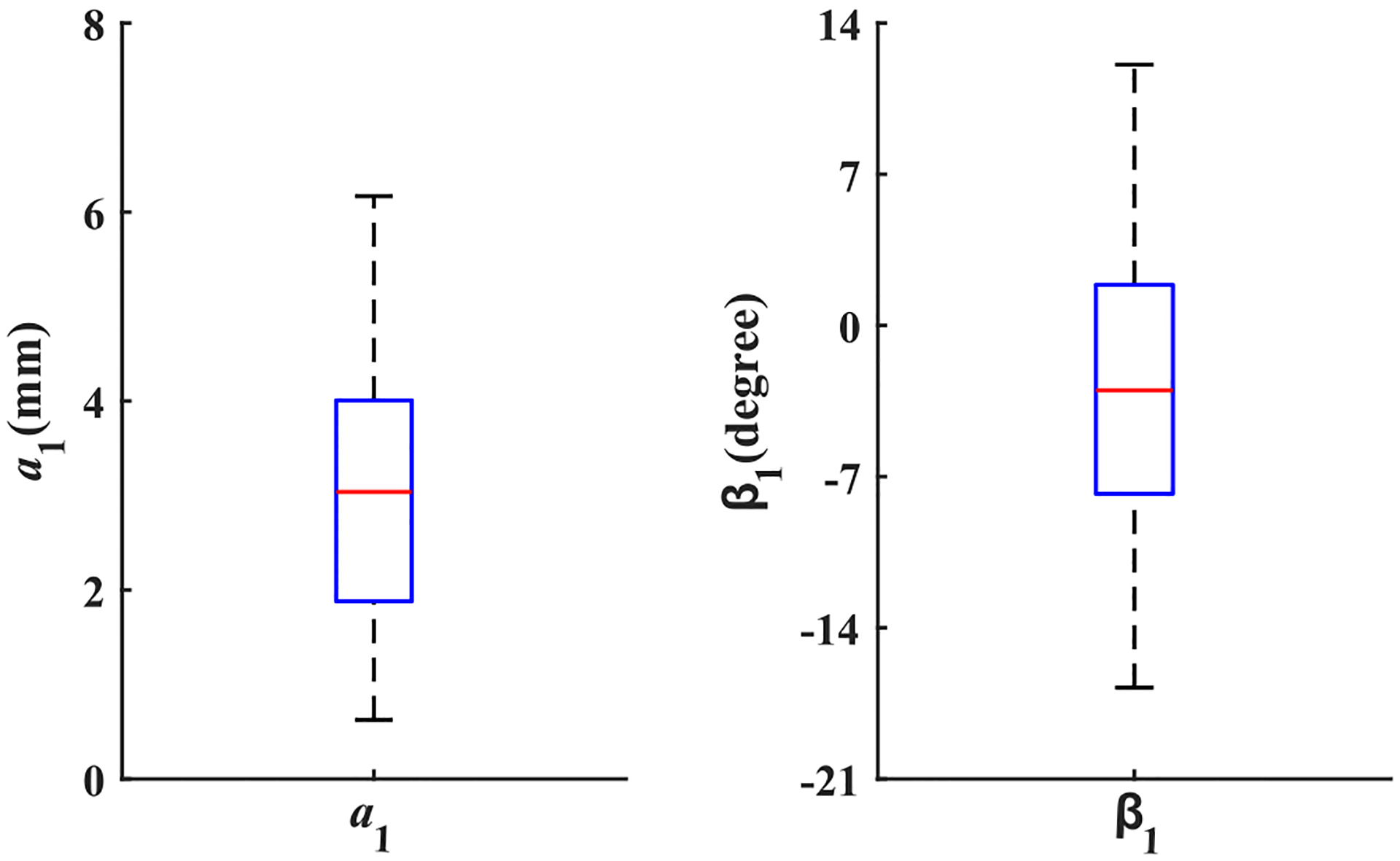
The *start point accuracy* and *lateral angle* of the needle in 42 cannulation trials.

The start point accuracy and the lateral angle of the needle in Phase 1 can be used to decide whether the fistula and its orientation were found accurately via palpation. Figure 7 shows the boxplot of the start point accuracy and the lateral angle of the needle in Phase 1 during the experiment. The radius of the fistula is 7 mm. All the values of the start point accuracy are less than 7 mm, which means that all the participants found the fistula. The positive value of the lateral angle shows that the insertion is toward the fistula, while the negative value of the lateral angle shows that the insertion is away from the fistula. The boxplot of the lateral angle shows that not all subjects found the fistula orientation correctly.

In addition to the start point accuracy and the lateral angle of the needle, the process metrics used in this study comprise time span (*t*), needle tip path length (PL), average velocity (*v*), average insertion angle (*α*) and the average rate of change of the insertion angle $\left({\dot{\alpha }}_{i}\right)$. A statistical analysis of process metrics divided into Phases 1 and 3 was conducted depending on the needle insertion subtask for each cannulation cycle. After testing for normality of data, for time span, path length, average velocity and average insertion angle, paired *t*-tests were employed while Mann-Whitney *U*-tests were used for the other process metrics.

(9) $$\left[\begin{array}{l} x\\ y \end{array}\right]=\left[\begin{array}{l} b_{1}\\ b_{2} \end{array}\right]+\left[\begin{array}{l} k_{11}\\ k_{12} \end{array}\right]\cdot t+\left[\begin{array}{l} k_{21}\\ k_{22} \end{array}\right]\cdot PL+\left[\begin{array}{l} k_{31}\\ k_{32} \end{array}\right]\cdot v+\left[\begin{array}{l} k_{41}\\ k_{42} \end{array}\right]\cdot \alpha +\left[\begin{array}{c} k_{51}\\ k_{52} \end{array}\right]\cdot \dot{\alpha }+\left[\begin{array}{c} k_{61}\\ k_{62} \end{array}\right]\cdot a+\left[\begin{array}{c} k_{71}\\ k_{72} \end{array}\right]\cdot \beta$$

The results of the statistical analysis for process metrics in Phases 1 and 3 are presented in Table 2. From the results, all the *p*-values of the process metrics except the start point accuracy of the needle are less than 0.05, indicating significant differences between Phases 1 and 3 insertion behavior. Time taken in Phase 3 was significantly shorter than that in Phase 1 (3.02 s vs. 4.36 s) while path length also showed a similar decrease (15.36 mm vs. 22.61 mm). Average needle insertion velocity, which may also be related to insertion force, also was significantly different in both phases (1.08 mm/s in Phase 1 vs. 1.42 mm/s in Phase 3). In addition, angle metrics — average insertion angle and average rate of change of insertion angle — also demonstrated significant differences between Phases 1 and 3 (see Table 2). Lateral angle, a measure of the needle’s orientation with respect to the fistula, also demonstrated statistical difference (−3.39° in Phase 1 vs. −6.73° in Phase 3).

It appears that the start point accuracy (*a*) has limited value when used alone to distinguish between the behavior of Phases 1 and 3. However, as will be presented in the later subsection, *a* is valuable for predicting infiltration risk, an outcome metric.

Further, our results verify that users were following the prescriptions for needle insertion that direct users to “level out” the needle by lowering the insertion angle while advancing the needle further through the fistula. As such, the insertion angle and the rate of change of insertion angle in Phase 3 are significantly smaller than in Phase 1.

#### 3.1.2. Outcome metrics

**Fig. 8.**
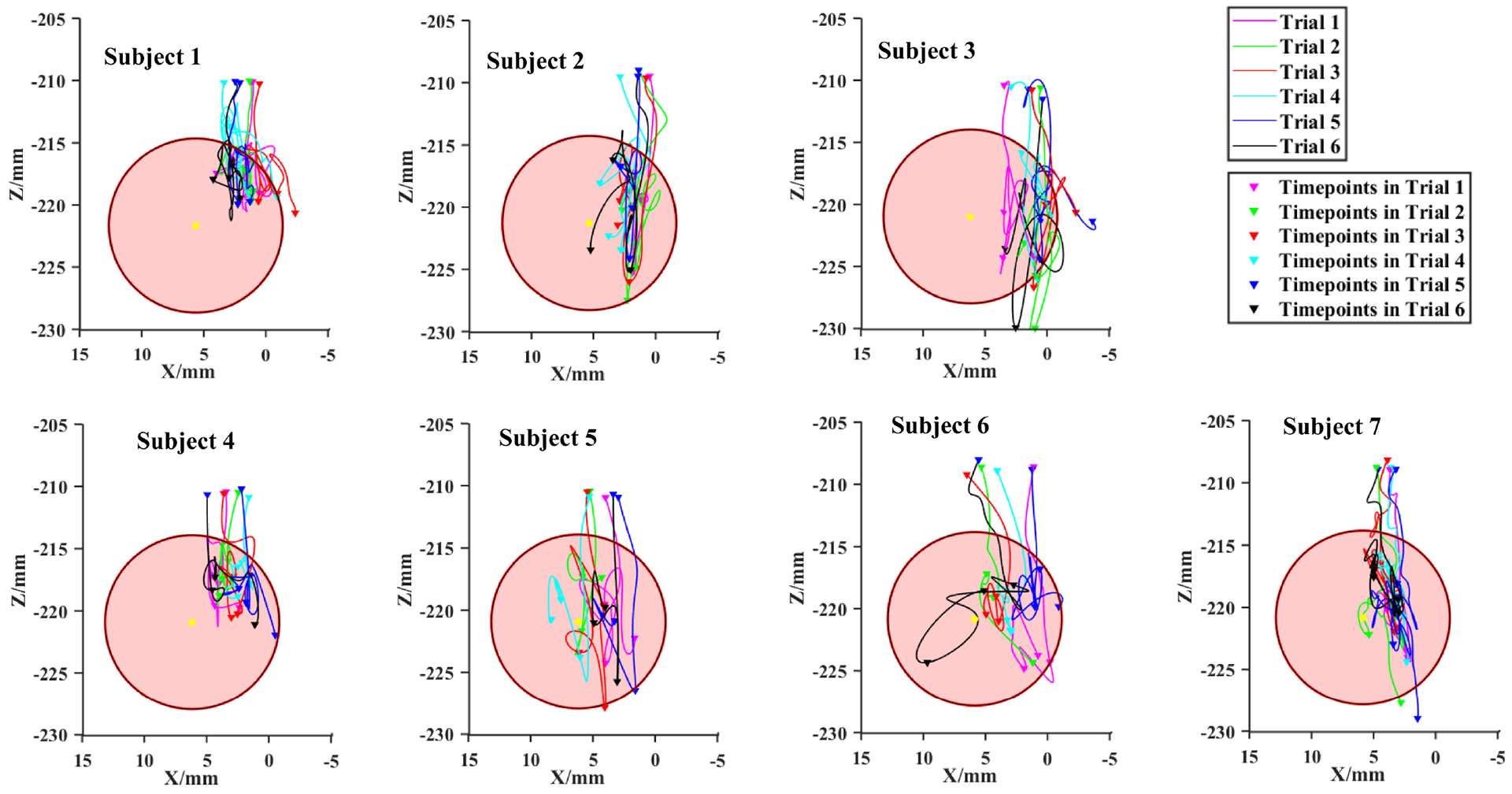
The 3D trajectories of the needle tip of 42 trials projected onto the fistula cross plane. *Notes*: Trajectories are segmented by time points marked with the triangle symbol. Any part of a trajectory lying outside the circle after the needle is inserted into the fistula for the first time implies infiltration.

Figure 8 demonstrates the projected 3D trajectories onto the cross-section of the fistula for all subjects, which provide evidence of needle infiltration. In each cannulation cycle, four small triangles indicate the segmentation of the needle insertion into the three phases. If a part of the projected curve of the 3D trajectory of the needle tip in the cross sectional plane of the fistula reaches beyond the circle (with radius = fistula radius = 7 mm) after the needle is inserted into the fistula, this indicates that infiltration has occurred. Based on this, the number of times infiltration occurred in each phase is presented in Table 3.

**Fig. 9.**
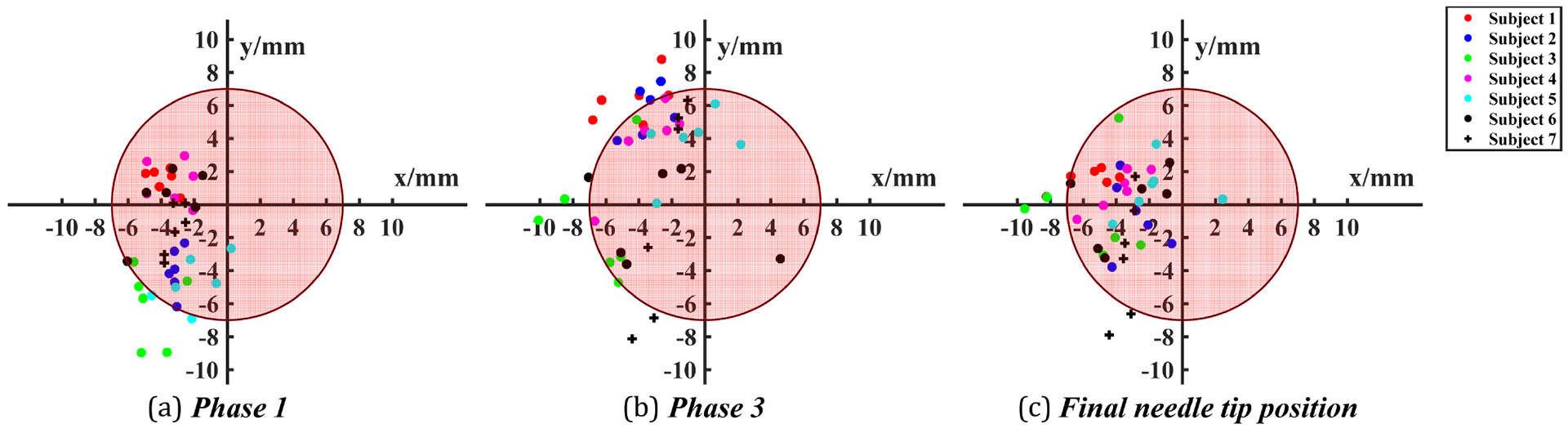
The infiltration risk point projected to the cross section of the fistula in Phases 1 and 3, and the final needle tip position projected to the cross section of the fistula.

Figures 9(a) and 9(b) plot the infiltration risk points in Phases 1 and 3 as per the definition. These points are a measure of the maximum distance that the needle has traveled beyond the fistula circumference. As can be noted from Table 3, there seem to be specific patterns in infiltration risk in Phases 1 and 3. In addition, we also examined the final needle tip position inside the fistula as this illustrates whether the needle rests in a secure location inside the fistula after needle insertion is completed. All final needle tip positions were projected onto the cross sectional plane and are presented in Fig. 9(c). Out of 42 trials, there were 6 trials where the needle tip rested outside the fistula, resulting in unsuccessful cannulation.

### 3.2. Process metrics predict outcome metrics

For calculating infiltration risk, we set the center of the fistula cross section plane as the origin (0, 0). The coordinates (*x*, *y*) are the 2D position of the needle tip in the cross sectional plane of the fistula at specific time points. As such, (*x*, *y*) was used to calculate the risk of infiltration. The results indicated that the outcome metric of infiltration risk can be predicted by phase-based process metrics using the multiple linear regression model (see Eq. (9)). Per Table 4, the regression model containing the metrics time, path length, average insertion angle, average rate of change of insertion angle, start point accuracy, and lateral angle were found to predict outcome metrics in the two phases.

For Phase 1, the *p*-values of path length, start point accuracy, and lateral angle are significant predictors of infiltration risk in the *x* or lateral direction (that is, infiltration of the sides of the fistula). In the *y*-direction, time, path length, average insertion angle and average rate of change of insertion angle are significant predictors of infiltration risk in the vertical direction (*top* or *bottom* infiltration). For Phase 3, path length, average rate of change of insertion angle, start point accuracy, and lateral angle were significant predictors in the *x*-direction. Because of the twin actions of leveling out and forwarding, the rate of change of angle was also found to contribute significantly to the prediction of infiltration risk in the lateral direction. In the vertical direction (*y*), time, path length, average insertion angle, start point accuracy and lateral angle were found to be significant predictors of infiltration risk.

To our knowledge, for the first time our results demonstrate that process metrics can be used to predict the risk of infiltration — an outcome metric — at the subtask level. Our results confirmed our initial hypothesis that different process metrics might impact needle movement in the lateral and vertical directions differently. In both Phases 1 and 3, time, path length, and average insertion angle significantly impacted (*p* < 0.05) the depth of the needle tip, i.e., the *y* value of the dependent variable. In addition, both start point accuracy and lateral angle are significant predictors of infiltration risk in the lateral direction. This detail makes intuitive sense given the geometrical relationship between needle trajectory with respect to fistula orientation.

## 4. Discussion

There is a recognized need in the medical education community for tools that enable competency-based education. Traditionally, medical training has relied heavily on on-the-job training where the number of years of experience was implicitly considered to correlate to skill level. However, this assumption is not necessarily true. Due to the increased focus on reducing medical error and ensuring patient safety, there is a demand for competency-based training. That is, performance based on validated skill metrics should be the accepted basis for assessing skill level. In light of this, several medical disciplines have implemented competency-based credentialing for medical professionals (e.g. the Fundamentals of Laparoscopic Skills curriculum) [25]. The work presented here aims to lay the foundation for medical educators to objectively and systematically assess needle insertion skills for vascular access.

To our knowledge, we have developed the first simulator for quantifying needle insertion skill during cannulation. Our simulator is capable of objectively measuring needle motion, angle, etc. via sensors incorporated into the system. Further, the parameters of AV fistulas, like skin thickness, depth of the fistula, diameter and curvature of the fistula can be easily changed in our simulator allowing for variety in training. This feature is typically not available in current “fake arms” for intravenous or HDC training. Fake arms also do not feature sensor-based assessment of skill — a critical need for competency-based skill assessment. In this work, we have demonstrated that our system is not only able to quantify skill, but also to segment critical aspects of the workflow by synchronizing video and EM tracking sensor data. As such, this system provides a basis for a precise and rich assessment of cannulation skills.

One of the most salient results from this study is the quantification of the novice subjects’ performance on this relatively easy cannulation task. For instance, the average insertion angle was about 55°, which is significantly higher than the prescribed 20–35° angle for fistulas. Further, the rate of needle infiltration was 35% in Phase 3; that is, one in three needle insertions resulted in an infiltration despite a very large and completely straight fistula in a simulator environment. These types of data can provide an objective basis for the training of novice cannulators in contrast with the absence of such data on “fake arms”.

An important result presented here pertains to the difference in the metrics during two primary phases of needle insertion. Most, if not all, previous studies that used sensors for needle-based skill assessment computed metrics for the whole needle insertion process (from puncture to completion). We posited that dividing the needle insertion procedure into phases will enable more meaningful skills assessment and provide greater specificity since the goals for these phases differ. For instance, the goal in Phase 1 is to obtain flashback, while the goal in Phase 3 is to secure the needle within the vascular access so as to allow for sustained blood withdrawal. Therefore, needle insertion skill is better measured at the finer resolution of phases in contrast to the start-to-finish insertion. Results from our study confirm the hypothesis that process metrics significantly differ in Phases 1 and 3. Consequently, needle insertion training in the future should consider incorporating phase-specific metrics for skill assessment.

In this work, we have also introduced the differentiation between outcome and process metrics for cannulation training. From a clinical perspective, outcome metrics are what ultimately matter since patient outcomes are directly correlated to procedural outcomes. In our case, infiltration of the needle outside the simulated blood vessel wall after puncture is considered an undesirable outcome measure. As presented in Figs. 8 and 9 and Table 3, our methods can objectively identify outcome metrics, namely, infiltration risk. We hypothesized that the outcome metric (infiltration) can be predicted by phase-specific process metrics. To our knowledge, this is the first time that the relationship between process metrics and outcome metrics has been explored. Results from our study demonstrate that process metrics are able to predict the outcome metric of risk of infiltration. This can be applied in the future for predicting the likelihood of a successful outcome from process metrics alone. In addition, our results also reveal that the process-outcome metric relationship is phase dependent.

Finally, we note several implications from this study for cannulation skills training. First, the current prescription for accurate and safe cannulation for AV fistulas is based primarily on needle angle (20–35°) [5]. However, this is hard to measure in the clinical setting. Also, following this guideline may help obtain flashback but has limited utility for preventing infiltration. In addition to this, the needle angle is dependent on the anatomical parameters of both the skin and the fistula (skin thickness, depth of fistula from skin, fistula diameter and curvature, etc.). Clinicians must be taught to adapt their needle angle as a function of these parameters. In our study, novice participants used angles greater than the prescribed range. However, a training regimen based on angles alone may prove to be insufficient for skills training. A more complete method will incorporate a suite of both process and outcome metrics based on objective sensor data as presented in this paper.

We wish to conclude this section by noting a few caveats. First, results presented in this study are preliminary in nature. Extensive validation is required to establish the metrics used here for skill training and assessment. We are taking steps towards this by further refining the simulator and planning to collect data from a larger set of clinicians along a broad spectrum of skill levels. In line with this, readers are also reminded that all subjects in this study were novice nursing students who were inexperienced in cannulation for vascular access. In the future, we will build upon this study by collecting data from both experts and novices for cannulation skills assessment.

## 5. Conclusion

In conclusion, we present a novel system for quantifying vascular access cannulation skill on a simulator outfitted with video and electromagnetic sensors. This system is capable of capturing synchronized video and motion data that is used for segmenting workflow as well as extracting phase-specific meaningful metrics for skill. Results from the study demonstrate that metrics are different for the two critical phases of needle insertion — flashback (Phase 1) and securing the needle (Phase 3) — indicating that training should be phase-specific. Further, for the first time, we present results that relate an outcome metric (risk of infiltration) to process metrics measured during a task. These results provide a solid foundation for more extensive research on objective, simulator-based cannulation skills training, which in turn could greatly enhance patient outcomes in vascular access if applied on a large scale.

## Figures and Tables

**Table 1.** Phase-based metrics extracted in each phase.

Name of phase	Outcome metrics	Process metrics
Insertion phase (Phase 1)	Infiltration risk (*x*_1_, *y*_1_)	*t*_1_, PL_1_, *v*_1_, *α*_1_, ${\dot{\alpha }}_{1}$, *a*_1_, *β*_1_
Rotation phase (Phase 2)	N/A	N/A
Leveling and forwarding phase (Phase 3)	Infiltration risk (*x*_3_, *y*_3_), and final needle tip position (*x*_*f*_, *y*_*f*_)	*t*_3_, PL_3_, *v*_3_, *α*_3_, ${\dot{\alpha }}_{3}$, *a*_3_, *β*_3_

**Table 2.** The statistical results of phase-based process metrics.

Name of metrics	Mean ± SD (Phase 1)	Mean ± SD (Phase 3)	*p*-value
Time *t*_*i*_ (s)	4.36 ± 3.18	3.02 ± 1.57	0.005*
Path length PL_*i*_ (mm)	22.61 ± 6.28	15.36 ± 5.12	< 0.001*
Average velocity *v*_*i*_ (mm/s)	1.08 ± 0.71	1.42 ± 1.02	0.04*
Average insertion angle *α*_*i*;_ (°)	54.45 ± 8.67	34.98 ± 6.62	< 0.001*
Average rate of change of insertion angle ${\dot{\alpha }}_{i}$ (°/s)	−5.12 ± 8.07	−7.62 ± 5.38	0.004*
Start point accuracy *a*_*i*_ (mm)	3.02 ± 1.41	3.05 ± 1.60	0.827
Lateral angle *β*_*i*_ (°)	−3.39 ± 6.56	−6.73 ± 15.06	0.037*

*Note*: Metrics with statistical significance (*p*-value < 0.05) are marked with*.

**Table 3.** Number of different types of error in Phases 1 and 3.

Infiltration count	Phase 1	Phase 3
*Bottom* infiltration	6	6
*Top* infiltration	3	9
*Total* infiltration	9	15

**Table 4.** The *p*-values of process metrics in Phases 1 and 3.

*p*-value		*t*	PL	*v*	*α*	$\dot{\alpha }$	*a*	*β*	*R*-square (%)
Phase 1	*x*	0.116	0.014*	0.475	0.212	0.874	*<* 0.001 *	*<* 0.001 *	89.02
	*y*	0.001*	0.014*	0.991	*<* 0.001 *	0.002*	0.052	0.683	68.01
Phase 3	*x*	0.049	0.003*	0.649	0.650	0.004*	< 0.001 *	0.009 *	66.95
	*y*	0.021*	0.020*	0.794	< 0.001 *	0.371	0.004*	0.005*	68.06

*Note*: Metrics with statistical significance (*p*-value < 0.05) are marked with*.

## References

[1] Ravik M, Havnes A and Bjørk IT, Conditions affecting the performance of peripheral vein cannulation during hospital placement: A case study, Nurs. Res. Pract 2017 (2017). 10.1155/2017/9748492 PMC5697389 29238617

[2] Helm RE, Klausner JD, Klemperer JD, Flint LM and Huang E, Accepted but unacceptable: Peripheral iv catheter failure, J. Infus. Nurs 38(3) (2015) 189–203. 25871866 10.1097/NAN.0000000000000100

[3] Keleekai NL, Schuster CA, Murray CL, King MA, Stahl BR, Labrozzi LJ, Gallucci S, LeClair MW and Glover KR, Improving nurses’ peripheral intravenous catheter insertion knowledge, confidence, and skills using a simulation-based blended learning program: A randomized trial, Simul. Healthc 11(6) (2016) 376. 27504890 10.1097/SIH.0000000000000186 PMC5345884

[4] Makary MA and Daniel M, Medical error — the third leading cause of death in the US, BMJ 353 (2016) i2139. 27143499 10.1136/bmj.i2139

[5] Brouwer DJ, Cannulation camp: Basic needle cannulation training for dialysis staff, D & T 40(10) (2011) 434–439.

[6] Dinwiddie LC, Ball L, Brouwer D, Doss-McQuitty S and Holland J, What nephrologists need to know about vascular access cannulation, Semin Dial. 26(3) (2013) 315–322. 23458148 10.1111/sdi.12069

[7] Parisotto MT, Schoder VU, Miriunis C, Grassmann AH, Scatizzi LP, Kaufmann P, Stopper A and Marcelli D, Cannulation technique influences arteriovenous fistula and graft survival, Kidney Int. 86(4) (2014) 790–797. 24717298 10.1038/ki.2014.96 PMC4184025

[8] Lee T, Barker J and Allon M, Needle infiltration of arteriovenous fistulae in hemodialysis: Risk factors and consequences, Am. J. Kidney Dis 47(6) (2006) 1020–1026. 16731297 10.1053/j.ajkd.2006.02.181

[9] Clinkard D, Moult E, Holden M, Davison C, Ungi T, Fichtinger G and McGraw R, Assessment of lumbar puncture skill in experts and nonexperts using checklists and quantitative tracking of needle trajectories: Implications for competency-based medical education, Teach Learn. Med 27(1) (2015) 51–56. 25584471 10.1080/10401334.2014.979184

[10] Yin MS, Haddawy P, Suebnukarn S and Rhienmora P, Automated outcome scoring in a virtual reality simulator for endodontic surgery, Comput. Methods Programs Biomed 153 (2018) 53–59. 29157461 10.1016/j.cmpb.2017.10.001

[11] Nguyen XA, Ljuhar D, Pacilli M, Nataraja RM and Chauhan S, Surgical skill levels: Classification and analysis using deep neural network model and motion signals, Comput. Methods Programs Biomed 177 (2019) 1–8. 31319938 10.1016/j.cmpb.2019.05.008

[12] Grantcharov TP, Bardram L, Funch-Jensen P and Rosenberg J, Learning curves and impact of previous operative experience on performance on a virtual reality simulator to test laparoscopic surgical skills, Am. J. Surg 185(2) (2003) 146–149. 12559445 10.1016/s0002-9610(02)01213-8

[13] Thijssen AS and Schijven MP, Contemporary virtual reality laparoscopy simulators: Quicksand or solid grounds for assessing surgical trainees?, Am. J. Surg 199(4) (2010) 529–541. 20080227 10.1016/j.amjsurg.2009.04.015

[14] Yeo CT , The effect of augmented reality training on percutaneous needle placement in spinal facet joint injections, IEEE Trans. Biomed. Eng 58(7) (2011) 2031–2037. 21435970 10.1109/TBME.2011.2132131

[15] Yeo CT, Davison C, Ungi T, Holden M, Fichtinger G and McGraw R, Examination of learning trajectories for simulated lumbar puncture training using hand motion analysis, Acad. Emerg. Med 22(10) (2015) 1187–1195. 26381528 10.1111/acem.12753

[16] Nasseri MA, Dean EC, Nair S, Eder M, Knoll A, Maier M and Lohmann CP, Clinical motion tracking and motion analysis during ophthalmic surgery using electromagnetic tracking system, 2012 5th Int. Conf. BioMedical Engineering and Informatics (IEEE, 2012), pp. 1058–1062.

[17] Chen H-E, Yovanoff MA, Pepley DF, Prabhu RS, Sonntag CC, Han DC, Moore JZ and Miller SR, Evaluating surgical resident needle insertion skill gains in central venous catheterization training, J. Surg. Res 233 (2019) 351–359. 30502270 10.1016/j.jss.2018.07.040 PMC6290357

[18] Forestier G, Lalys F, Riffaud L, Trelhu B and Jannin P, Classification of surgical processes using dynamic time warping, J. Biomed. Inform 45(2) (2012) 255–264. 22120773 10.1016/j.jbi.2011.11.002

[19] Holden MS, Ungi T, Sargent D, McGraw RC and Fichtinger G, Surgical motion characterization in simulated needle insertion procedures, in Proc. SPIE, Medical Imaging 2012: Image-Guided Procedures, Robotic Interventions, and Modeling, Vol. 8316 (International Society for Optics and Photonics, 2012), p. 83160W.

[20] Holden MS, Ungi T, Sargent D, McGraw RC, Chen EC, Ganapathy S, Peters TM and Fichtinger G, Feasibility of real-time workflow segmentation for tracked needle interventions, IEEE Trans. Biomed. Eng 61(6) (2014) 1720–1728. 24845282 10.1109/TBME.2014.2301635

[21] Dergachyova O, Bouget D, Huaulmé A, Morandi X and Jannin P, Automatic data-driven real-time segmentation and recognition of surgical workflow, Int. J. Comput. Assist. Radiol. Surg 11(6) (2016) 1081–1089. 26995598 10.1007/s11548-016-1371-x

[22] Hisey R, Ungi T, Holden M, Baum Z, Keri Z, McCallum C, Howes DW and Fichtinger G, Real-time workflow detection using webcam video for providing real-time feedback in central venous catheterization training, in Proc. SPIE, Medical Imaging 2018: Image-Guided Procedures, Robotic Interventions, and Modeling, Vol. 10576 (International Society for Optics and Photonics, 2018), p. 1057620.

[23] Reiley CE and Hager GD, Task versus subtask surgical skill evaluation of robotic minimally invasive surgery, in Int. Conf. MICCAI (Springer, 2009), pp. 435–442. 10.1007/978-3-642-04268-3_54 20426017

[24] Kil I, Groff RE and Singapogu RB, Assessment of open surgery suturing skill — I: Simulator platform, force-based and motion-based metrics, IEEE Trans. Biomed. Eng. (submitted) 10.3389/fmed.2022.897219 PMC9468321 36111107

[25] Gallagher AG and O’Sullivan GC, Fundamentals of Surgical Simulation: Principles and Practice, 1st edn. (Springer, 2011).

